# Centaurine

**Published:** 1846-03

**Authors:** 


*Centaurine.*—M. Gennaro Galani states that he has extracted the active principle of the lesser centaury, and finds it a good succedaneum for quinine.—*Ibid.*

